# The association between fatty liver index and onset of diabetes: secondary analysis of a population-based cohort study

**DOI:** 10.1186/s12889-023-15442-z

**Published:** 2023-04-11

**Authors:** Yanqiong Zhu, Haofei Hu, Yumei Wu, Yinhua Rao, Qixiang Li, Xuehui Duan, Guopeng Yao, Hekun Yin, Ziyi Luo

**Affiliations:** 1 Department of Gastroenterology, Jiangmen Central Hospital, No.23 Haibang Street, Pengjiang District, Jiangmen, 529030 Guangdong Province China; 2 Department of Nephrology, The First Affiliated Hospital of Shenzhen University, Shenzhen, 518000 Guangdong Province China; 3 Department of Nephrology, Shenzhen Second People’s Hospital, Shenzhen, 518000 Guangdong Province China; 4 Shenzhen University Health Science Center, Shenzhen, 518000 Guangdong Province China; 5 Department of Gastroenterology, Shenzhen Third People’s Hospital, No.29 Bulan Road, Longgang District, Shenzhen, 518000 Guangdong Province China; 6 Department of Gastroenterology, Shenzhen People’s Hospital Longhua Branch, Guangdong Province, 518000 China

**Keywords:** Fatty liver index, Incident diabetes, Association

## Abstract

**Background:**

According to research, the fatty liver index (FLI) is associated with diabetes. However, few studies have been conducted to investigate the relationship between FLI and diabetes risk from various perspectives. This study comprehensively investigated the relationship between FLI and incident diabetes in a large Japanese population.

**Methods:**

This retrospective cohort study included 14,280 participants from Murakami Memorial Hospital in Japan from 2004 to 2015. The independent and dependent variables are FLI and risk of type 2 diabetes mellitus (T2DM), respectively. To examine the link between FLI and incident T2DM, Cox proportional-hazards regression was employed. In addition, we performed a number of sensitivity studies to guarantee the validity of the results. Moreover, we conducted subgroup analyses.

**Results:**

After adjusting covariates, the results showed that FLI was positively associated with the risk of T2DM (HR = 1.019, 95%CI: 1.012, 1.025). Additionally, the sensitivity analysis showed how reliable the outcomes were. And a stronger association between FLI and incident T2DM was observed in the regular exercisers (HR = 1.036, 95%CI: 1.019–1.053, *P* < 0.0001) and the population without ethanol consumption (HR = 1.028, 95%CI: 1.017–1.039, *P* < 0.0001). Besides, receiver operating characteristic (ROC) curve analysis showed that FLI was better than waist circumference, triglycerides, body mass index, and gamma-glutamyl transferase in predicting incident T2DM.

**Conclusion:**

FLI is positively associated with incident T2DM.

**Supplementary Information:**

The online version contains supplementary material available at 10.1186/s12889-023-15442-z.

## Background

Diabetes is one of the twenty-first century's fastest-rising diseases. According to the International Diabetes Federation, 537 million people were diagnosed with diabetes mellitus worldwide in 2021, with the figure anticipated to rise to 783 million by 2045 [1]. As the most common chronic disease globally, diabetes and its complications significantly impact individuals and their families, national economies, and health systems. Diabetes was expected to cost the world USD 966 billion in direct health spending in 2021 [2]. Sadly, the mortality rate related to type 2 diabetes mellitus (T2DM) complications was high, and it was estimated that more than 6.7 million adults aged 20–79 would die from diabetes in 2021 worldwide [1]. Therefore, it is critical to better understand the diabetes risk factors that can be employed for diabetes prevention and screening.

Numerous studies have revealed that fatty liver is a risk factor for developing diabetes [3–6]. Nonalcoholic fatty liver disease (NAFLD) is driven by ectopic fat accumulation in the liver. Diagnosis of NAFLD is defined as the presence of hepatic steatosis, ballooning, and lobular inflammation with or without fibrosis [7]. Previous studies have shown that hepatic steatosis could instigate liver injury, inflammation, and insulin resistance (IR) [8, 9]. IR is a determining factor in the pathophysiology of T2DM [10]. The gold standard to diagnose hepatic steatosis is liver biopsy, but it has the limitations such as sampling variability, invasive nature, and high cost. Therefore, numerous non-invasive methods are used for diagnosis, including serum markers or imaging modalities such as ultrasound [11]. Fatty liver index (FLI) is a surrogate marker of hepatic steatosis, which is estimated from a formula that includes waist circumference (WC), body mass index (BMI), gamma-glutamyltransferase (GGT), and triglycerides (TG) [12]. This index could replace ultrasonography to diagnose the extent of hepatic steatosis and is a valid predictor of NAFLD [13]. Several studies have previously observed that FLI was a predictor of diabetes development [5, 13–16]. A meta-analysis [16] identified a positive association between FLI and the risk of diabetes mellitus incidence. However, because the majority of these studies did not use subgroup analysis, it was impossible to understand differences in the relationship between FLI and diabetes in different populations. Furthermore, the value of baseline FLI in predicting future diabetes occurrence, the optimal FLI threshold, and the sensitivity and specificity of predicting diabetes all require further investigation. In summary, more research was needed on the relationships between FLI and incident diabetes, such as the differences in the relationship between the two in different populations, the specific predictive value of FLI for diabetes, and so on. Therefore, this study aimed to comprehensively assess the relationship between FLI and incident diabetes in a large cohort of Japanese adults.

## Methods

### Data source and participants

The 'DATADRYAD' database, which was accessible at www.Datadryad.org and offered free raw data downloads, was used to collect the data. In this study, we used the Dryad data package based on the Dryad Terms of Service [17] (Dryad data package: Takuro Okamura et al. (2018) Data from: Ectopic fat obesity presents the greatest risk for incident type 2 diabetes: a population-based longitudinal study. Dryad Digital Repository. https://doi.org/10.1038/s41366-018-0076-3). Variables contained in the database file were as follows: sex, age, BMI, diastolic blood pressure (DBP), WC, systolic blood pressure (SBP), glycated hemoglobin, total cholesterol (TC), fasting plasma glucose (FPG), high-density lipoprotein cholesterol (HDL-C), GGT, ethanol consumption, alanine aminotransferase (ALT), TG, aspartate aminotransferase (AST), smoking status, fatty liver, exercise, follow-up days and censor of T2DM during follow up. The original study's author waived all copyright and associated privileges for this data. As a result, we were able to use these data without violating the authors' rights for our secondary analysis. This study was performed according to the Declaration of Helsinki. The Murakami Memorial Hospital Ethics Committee approved this study, and all subjects provided informed permission [17].

The original data were gathered from the database of Murakami Memorial Hospital in Japan's NAGALA (NAfld in the Gifu Area, Longitudinal Analysis) [17]. In order to minimize selection bias, the participants were collected consecutively from Murakami Memorial Hospital in Japan. Their identity information was encoded into an untraceable code to ensure the participants’ privacy. Inclusion criteria were: participants who took the physical test between 2004 and 2015, and had completed at least two physical examinations. The exclusion criteria were as follows in the original research: (1) participants diagnosed with type 2 diabetes (*n* = 323) or with fasting plasma glucose was over 6.1 mmol/L at baseline (*n* = 808), (2) participants with known liver disease, such as hepatitis B or C virus (*n* = 416), (3) anyone who took any medication at baseline examination (*n* = 2321), (4) those who drank heavily (ethanol intake greater than 60 g/day for men and 40 g/day for women) (*n* = 739), (5) participants with a missed value of covariates, including abdominal ultrasonography, exercise, alcohol intake or height (*n* = 863) [17]. And then, FLI was calculated as mentioned by Bedogni et al. [12]: FLI = 100/(1 + e^−z^); z = 0.953 × ln[TG (mg/dL)] + 0.139 × BMI (kg/m^2^) + 0.718 × ln[GGT (U/L)] + 0.053 × WC (cm)-15.745. For this further study, we excluded men with ethanol consumption over 30 g/day and women with ethanol consumption over 20 g/day (*n* = 1184).

### Study design and measurement of variables

The design of the study has been recorded elsewhere [17]. As mentioned in the NAGALA study, this study's clinical baseline information was collected through a standardized self-administered questionnaire, including physical activity, alcohol, smoking habits, and medical history. Ethanol consumption was evaluated by the mean ethanol intake of participants per week during the prior month. Smoking status was categorized into current smoker, ex-smoker, or non-smoker. To categorize participants into non- or regular exercisers, participants’ recreational and sports activities were investigated. Regular exercisers were defined as those who reported any exercise more than once a week [18]. BMI was computed as follows: BMI (kg/m^2)^ = body weight (kg)/height^2^ (m^2^). After a night of fasting, venous blood was collected for hematological indicators testing, including TC, GGT, TG, ALT, HDL-C, AST, glycated hemoglobin, and FPG. Fatty liver was detected by the findings of abdominal ultrasonography conducted by competent technicians [19]. Finally, FLI was the target-independent variable obtained at baseline, calculated as Bedogni et al. described [12]. Besides, the dependent variable was incident T2DM acquired during follow-up. Because it was a retrospective study, it reduced the possibility of observation and selection bias.

### Ascertainment of incident T2DM

T2DM was defined as fasting plasma glucose ≥ 7 mmol/L, glycated hemoglobin ≥ 6.5%, or self-reported during the follow-up period. The patients would be reviewed on the day of the diagnosis of T2DM or the day of the last visit, whichever comes first [20]. Participants who were unable to be reached for follow-up would still be included in the study [21].

### Statistical analysis

First, we dealt with missing values of covariables. In the present study, only TG had missing values. The number of participants due to missing TG values is only 11 (0.07%), so we used the average of TG to impute the missing values [22].

Second, we stratified the participants by quartiles of FLI. Categorical variables were expressed as percentages of a particular group, while continuous variables with normal and skewed distributions were expressed as means with standard deviations or medians with interquartile ranges. Next, the Chi-square, Kruskal–Wallis test, and one-way ANOVA were used for the four groups' categorical, skewed continuous, and normal continuous variables. Person-years of follow-up were computed from the date of the baseline interview until the occurrence of T2DM or the date of the follow-up interview [23]. The incidence rates were given in terms of cumulative incidence and person-year incidence [24]. The Kaplan–Meier method was used to calculate survival estimates and time-to-event variables. A log-rank test was employed to assess the Kaplan–Meier survival probability among FLI groups [25].

Third, and foremost, the proportion hypothesis was examined. The Cox proportional hazards regression model was then used to estimate the risk ratios (HRs) and 95% confidence intervals (CIs) of incident T2DM after meeting the proportion criteria. The results of unadjusted, minimally adjusted ( SBP, gender, smoking status, DBP, age, ethanol consumption, and exercise), and fully adjusted ( DBP, ALT, smoking status, SBP, exercise, AST, ethanol consumption, age, glycated hemoglobin, TC, gender, HDL-C, fatty liver) analyses were simultaneously shown based on the STROBE statement [21]. When the covariances were included in the model and the hazard ratio changed by 10% or more, we made the necessary adjustments [26].

Additionally, we conducted a series of sensitivity analyses to ensure the robustness of the data analysis [27]. FLI was transformed into a categorical variable, and the *P*-value for the trend was determined. The goal of the test was to confirm the results of considering FLI as a continuous variable and to examine the probability of non-linearity. We excluded participants with fatty liver or any alcohol consumers in other sensitivity analyses. Besides, in order to guarantee the reliability of the results, we also employed generalized additive models (GAM) to include the continuity covariate as a curve into the equation [28]. Additionally, by computing E-values, we investigated the possibility of unmeasured confounding between FLI and incident T2DM [29].

Fourth, we stratified Cox proportional hazard models to investigate the robustness of the results across different subgroups (SBP, DBP, FPG, exercise, age, fatty liver, ethanol consumption, sex, smoking status). Firstly, we converted the continuous variable SBP (< 140 mmHg, ≥ 140 mmHg), DBP (< 90 mmHg, ≥ 90 mmHg), age (< 30, 30 to 40, 40 to 50, 50 to 60, ≥ 60) [30], ethanol consumption (= 0 g/week, > 0 g/week) to a categorical variable based on the clinical cut point while converted FPG based on binary. Secondly, in addition to the stratification factor itself, we adjusted each stratification for all factors (DBP, sex, ALT, SBP, AST, fatty liver, glycated hemoglobin, HDL-C, ethanol consumption, age, smoking status, TC, and exercise). Lastly, the likelihood ratio test was used to test for interaction in models with and without interaction terms [31, 32].

Finally, the ability of FLI, WC, TG, BMI, and GGT to predict the risk of T2DM was estimated using a receiver operating characteristic (ROC) curve. We also calculated net reclassification improvement (NRI) and integrated discrimination improvement (IDI) to compare the predictive values among different models.

The statistical software programs R (http://www.R-project.org, The R Foundation) and Empower-Stats (http://www.empowerstats.com, X&Y Solutions, Inc., Boston, MA) were used for all these studies. P values that were less than 0.05 (two-sided) were regarded as statistically significant.

## Results

**Fig. 1:**
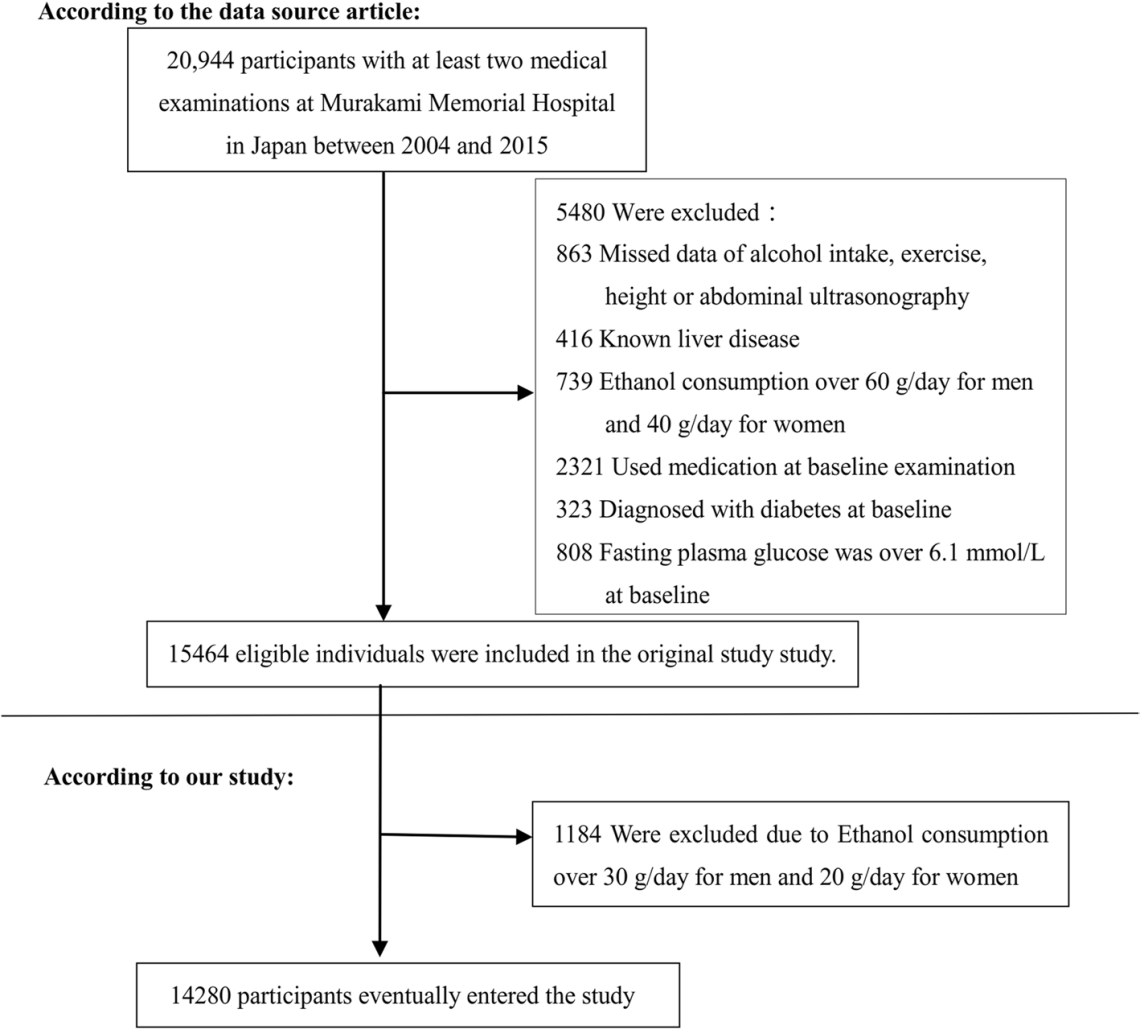
Flowchart of study participants

Initially, the study recruited 20,944 participants; afterward, 6,664 participants were excluded, and 14,280 persons (47.90% women and 52.10% men) remained for data analysis (Fig. 1). The average age was 43.53 ± 8.89 years old. During a mean follow-up period of 5.38 years, 324 (2.27%) individuals developed T2DM. The mean FLI was 12.61 ± 16.43, and the mean FPG was 5.15 ± 0.41 mmol/L.

### Baseline characteristics of the study participants

We assigned participants into subgroups using FLI quartiles (< 2.19,2.19–5.70,5.70–15.97, ≥ 15.97) and showed baseline characteristics of the total population in Table 1. In the highest FLI group, people generally had higher BMI, age, DBP, SBP, ethanol consumption, WC, AST, GGT, ALT, TG, TC, glycated hemoglobin, FPG, and higher rates of current smokers and ex-smokers. Besides, the group (FLI ≥ 15.97) had a higher proportion of men and fatty liver.

**Table 1:** The Baseline Characteristics of participants presented by quartiles of FLI

FLI group	Q1(< 2.19)	Q2(2.19–5.70)	Q3(5.70–15.97)	Q4(≥ 15.97)	*P*-value
Participants	3570	3570	3570	3570	
Age, years	40.29 ± 8.22	43.56 ± 8.79	45.16 ± 9.02	45.12 ± 8.62	< 0.001
Ethanol consumption, g/week	1.00 (0.00–4.47)	1.00 (0.00–31.59)	1.00 (0.00–60.00)	12.00 (1.00–84.00)	< 0.001
BMI, kg/m^2^	19.04 ± 1.56	20.93 ± 1.60	22.69 ± 1.78	25.61 ± 2.80	< 0.001
WC, cm	66.43 ± 4.61	72.89 ± 4.60	78.76 ± 4.60	86.70 ± 6.68	< 0.001
ALT, IU/L	13.00 (10.00–16.00)	14.00(12.00–18.00)	18.00(14.00–23.00)	25.00(19.00–35.00)	< 0.001
AST, IU/L	16.00 (13.00–19.00)	16.00(13.00–19.00)	17.00(14.00–21.00)	20.00(16.00–25.00)	< 0.001
GGT, IU/L	11.00 (9.00–13.00)	13.00(10.00–16.00)	16.00(13.00–21.00)	25.00(18.00–37.00)	< 0.001
HDL-C, mmol/L	1.72 ± 0.37	1.57 ± 0.38	1.38 ± 0.34	1.17 ± 0.28	< 0.001
TC, mmol/L	4.76 ± 0.78	5.03 ± 0.82	5.22 ± 0.84	5.49 ± 0.86	< 0.001
TG, mmol/L	0.42 (0.32–0.54)	0.62 (0.49–0.79)	0.85 (0.65–1.10)	1.37 (1.00–1.86)	< 0.001
Glycated hemoglobin, %	5.11 ± 0.30	5.16 ± 0.31	5.19 ± 0.32	5.25 ± 0.34	< 0.001
FPG, mmol/L	4.90 ± 0.38	5.07 ± 0.38	5.24 ± 0.36	5.38 ± 0.36	< 0.001
SBP, mmHg	104.91 ± 12.00	110.83 ± 12.69	116.67 ± 13.27	123.43 ± 14.50	< 0.001
DBP, mmHg	64.84 ± 8.27	68.78 ± 9.05	73.00 ± 9.33	77.95 ± 9.97	< 0.001
Male, n(%)	517 (14.48%)	1462 (40.95%)	2429 (68.04%)	3032 (84.93%)	< 0.001
fatty liver, n(%)	11 (0.31%)	110 (3.08%)	532 (14.90%)	1862 (52.16%)	< 0.001
Regular exercisers, n(%)	590 (16.53%)	690 (19.33%)	647 (18.12%)	549 (15.38%)	< 0.001
Smoking status					< 0.001
Non-smoker	2956 (82.80%)	2452 (68.68%)	1846 (51.71%)	1497 (41.93%)	
Ex-smoker	290 (8.12%)	523 (14.65%)	816 (22.86%)	943 (26.41%)	
Current smoker	324 (9.08%)	595 (16.67%)	908 (25.43%)	1130 (31.65%)	

Values are n(%) or mean ± SD or medians (quartiles)

*ALT* Alanine aminotransferase, *AST* Aspartate aminotransferase, *BMI* Body mass index, *DBP* Diastolic blood pressure, *FLI* Fatty liver index, *FPG* Fasting plasma glucose, *GGT* Gammaglutamyltransferase, *HDL-C* High-density lipoprotein cholesterol, *SBP* Systolic blood pressure, *TC* Total cholesterol, *TG* Triglyceride, *WC* Waist circumference

**Fig. 2:**
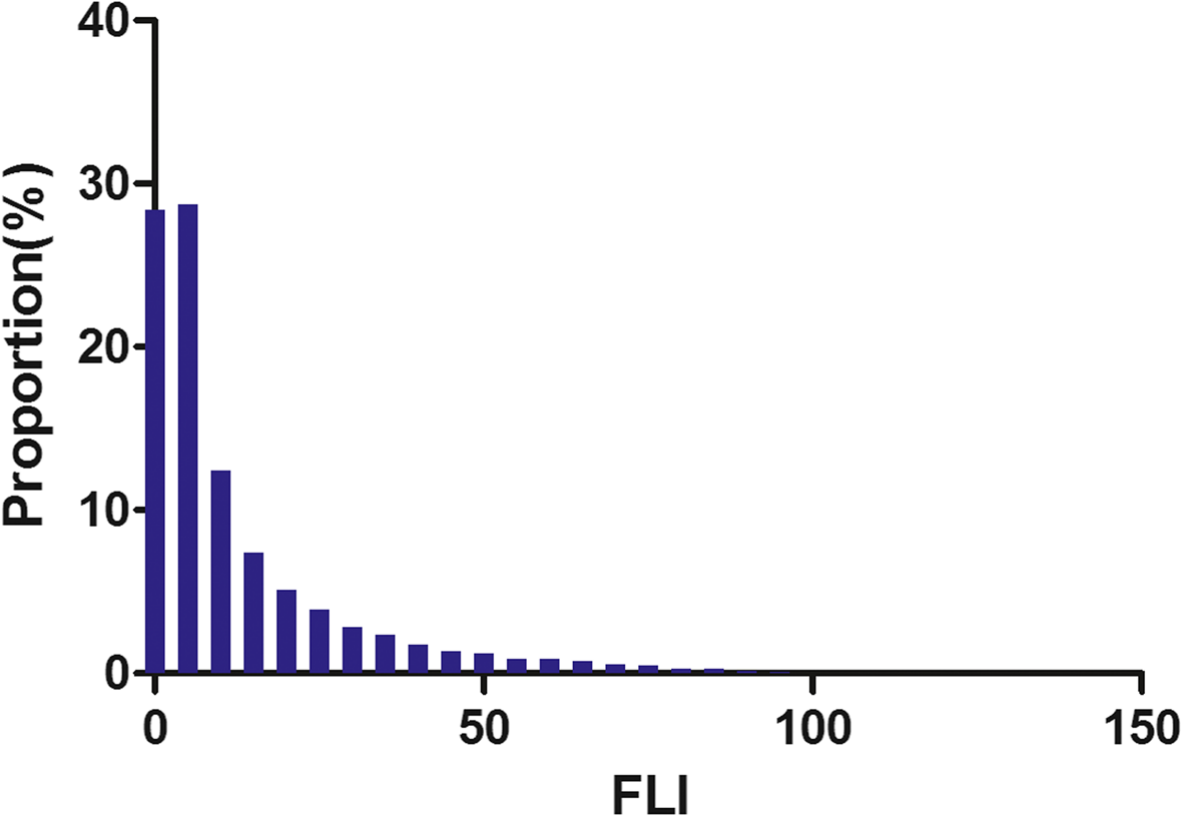
Distribution of FLI. It presented a skewed distribution while being in the range from 0.10 to 99.13

**Fig. 3:**
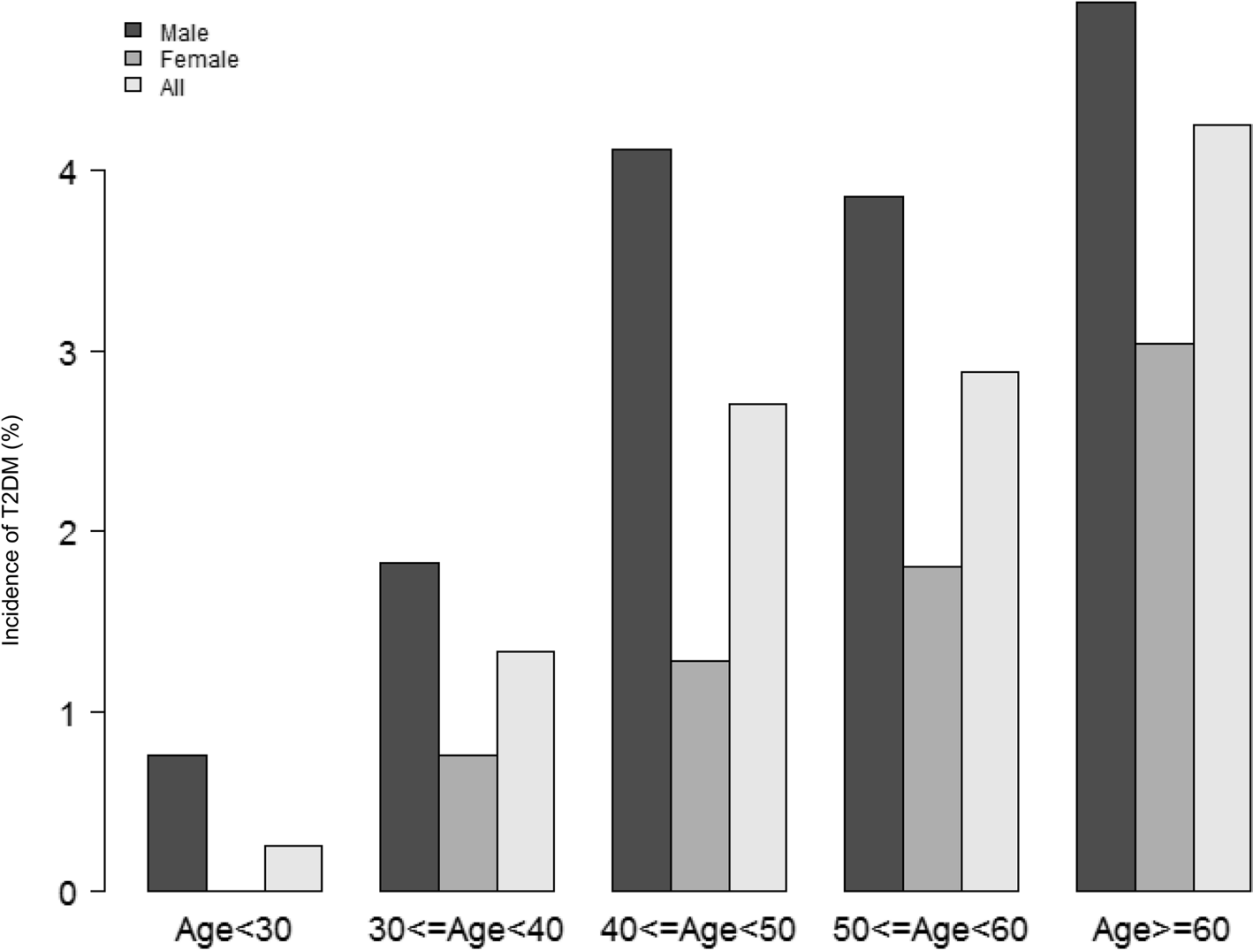
T2DM incidence of age stratification by 10 intervals

Figure 2 showed the distribution of FLI levels. It had a skewed distribution and was in the range of 0.10 to 99.13. This result also showed that the majority of the FLI was less than 60. Participants were separated into two groups based on whether or not they developed T2DM in the future. Figure S1 depicts the FLI values in the two groups. The results showed that the distribution level of FLI was higher in the T2DM group, whereas it was comparatively low in the T2DM-free group. Male individuals had a greater incidence rate of T2DM than female subjects in age stratification by ten intervals, regardless of age group (Fig. 3). We also observed that the incidence of T2DM increased with age in female participants.

### The incidence rate of incident T2DM

According to Table 2, 324 people had T2DM overall. All individuals' combined incidence rate was 375.23 per 100,000 person-years. Specifically, the incidence rates of the four FLI groups were 68.39, 122.69, 328.75, and 980.35 per 100,000 person-years, respectively. Participants with high FLI had a greater cumulative incidence (*P* < 0.001 for trend) than those with low FLI. The cumulative incidence of total incident T2DM and each FLI group was 2.269% (2.025%-2.513%), 0.420% (0.208%-0.632%), 0.728% (0.449%-1.007%), 1.989% (1.531%-2.447%), and 5.938% (5.163%-6.714%), respectively.

**Table 2:** Incidence rate of incident T2DM presented by quartiles of FLI

FLI	Participants(n)	T2DM events(n)	Cumulative incidence(95% CI)(%)	Per 100,000 person-year
Total	14,280	324	2.269 (2.025–2.513)	375.23
Q1	3570	15	0.420 (0.208–0.632)	68.39
Q2	3570	26	0.728 (0.449–1.007)	122.69
Q3	3570	71	1.989 (1.531–2.447)	328.75
Q4	3570	212	5.938 (5.163–6.714)	980.35
P for trend			< 0.001	

*FLI* Fatty liver index, *T2DM* Type 2 diabetes mellitus

### Univariate analysis

The results of the univariate analysis were shown in Table 3. By univariate Cox proportional hazard model, we found that exercise and ethanol consumption were not significantly associated with T2DM. We also found that HDL-C was negatively associated with incident T2DM. Univariate analysis illustrated that age, BMI, WC, ALT, SBP, GGT, glycated hemoglobin, TG, FPG, TC, AST, and DBP were positively associated with the risk of T2DM. Meanwhile, men, current and ex-smokers, and participants with fatty liver had a higher risk of developing T2DM.

**Table 3:** The results of univariate Cox proportional risk regression model for riks factors of T2DM

Variable	Statistics	HR (95%CI)	*P*value	
Age, years	43.533 ± 8.891	1.053 (1.040, 1.067)	< 0.00001	
BMI, kg/m^2^	22.068 ± 3.137	1.254 (1.227, 1.281)	< 0.00001	
WC, cm	76.196 ± 9.100	1.098 (1.087, 1.109)	< 0.00001	
ALT, IU/L	16.000 (12.000–23.000)	1.006 (1.005, 1.007)	< 0.00001	
AST, IU/L	18.227 ± 8.662	1.008 (1.006, 1.010)	< 0.00001	
GGT, IU/L	15.000 (11.000–21.000)	1.013 (1.010, 1.015)	< 0.00001	
HDL-C, mmol/L	1.459 ± 0.402	0.131 (0.094, 0.184)	< 0.00001	
TC, mmol/L	5.124 ± 0.868	1.529 (1.368, 1.709)	< 0.00001	
TG, mmol/L	0.723 (0.485–1.095)	1.846 (1.721, 1.979)	< 0.00001	
SBP, mmHg	113.961 ± 14.833	1.034 (1.028, 1.040)	< 0.00001	
DBP, mmHg	71.141 ± 10.391	1.051 (1.042, 1.061)	< 0.00001	
FLI	5.693 (2.191–15.962)	1.041 (1.038, 1.045)	< 0.00001	
FPG, mmol/L	5.148 ± 0.412	23.051 (16.736, 31.747)	< 0.00001	
Glycated hemoglobin, %	5.178 ± 0.321	54.893 (38.899, 77.461)	< 0.00001	
Ethanol consumption, g/week	1.000 (0.000–36.000)	1.000 (0.998, 1.002)	0.84232	
Sex				
Female	6840 (47.899%)	1.0		
Male	7440 (52.101%)	2.426 (1.892, 3.110)		< 0.00001
Fatty liver				
No	11,765 (82.388%)	1.0		
Yes	2515 (17.612%)	8.067 (6.438, 10.108)		< 0.00001
Regular exercisers				
No	11,804 (82.661%)	1.0		
Yes	2476 (17.339%)	0.802 (0.587, 1.095)		0.16493
Smoking status				
Non-smoker	8751 (61.282%)	1.0		
Ex-smoker	2572 (18.011%)	1.675 (1.248, 2.247)		0.00059
Current smoker	2957 (20.707%)	2.547 (1.995, 3.252)		< 0.00001

Values are n(%) or mean ± SD or medians (quartiles)

*ALT* Alanine aminotransferase, *AST* Aspartate aminotransferase, *BMI* Body mass index, *DBP* Diastolic blood pressure, *FLI* Fatty liver index, *FPG* Fasting plasma glucose, *GGT* Gammaglutamyltransferase, *HDL-C* High density lipoprotein cholesterol, *SBP* Systolic blood pressure, *TC* Total cholesterol, *TG* Triglyceride, *T2DM* Type 2 diabetes mellitus, *WC* Waist circumference

**Fig. 4:**
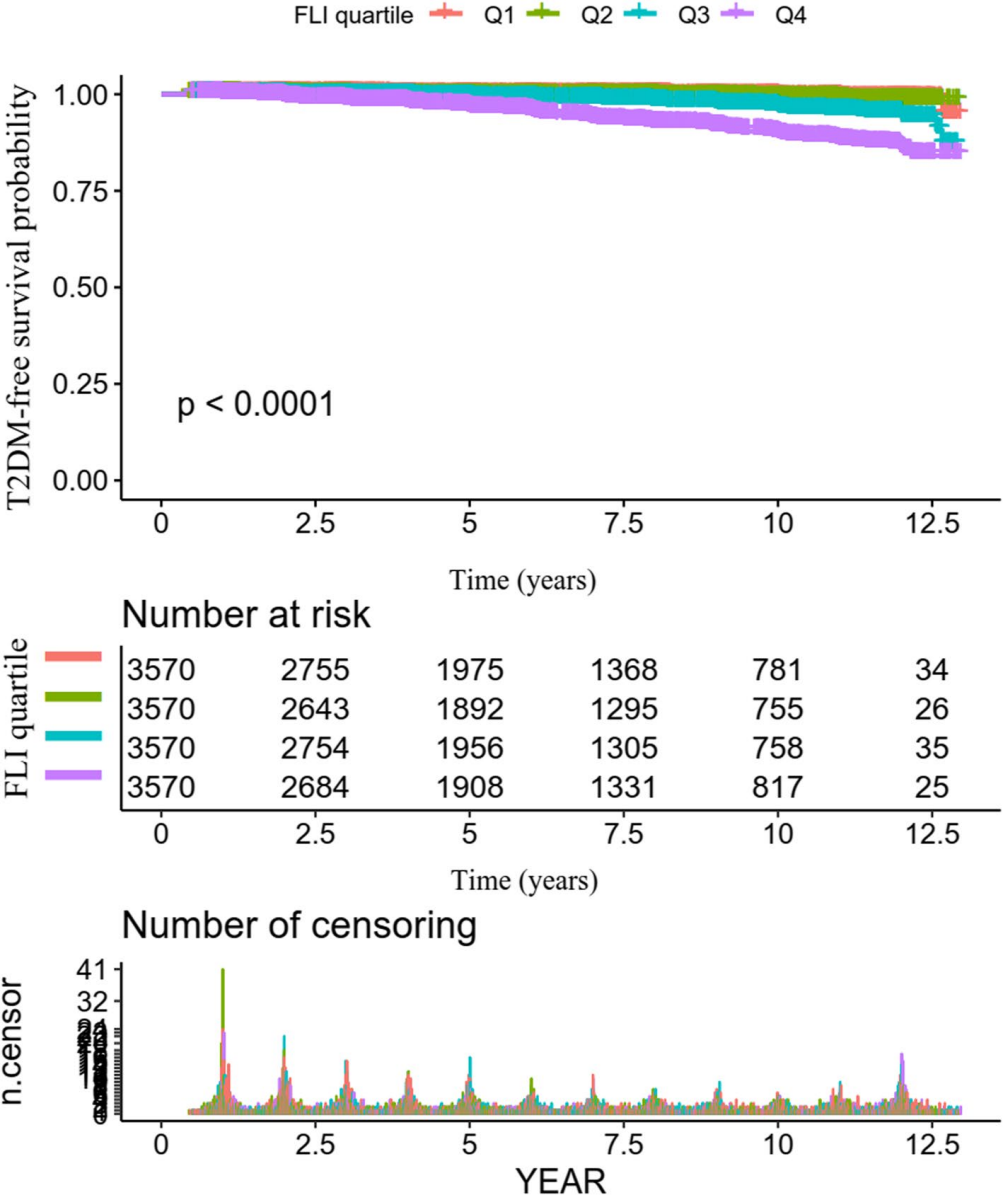
Kaplan–Meier event-free survival curve. Kaplan–Meier event-free survival curve. Kaplan–Meier analysis of incident T2DM-free survival based on FLI groups (log-rank, *P* < 0.0001)

Figure 4 displayed the Kaplan–Meier survival curves for the probability of T2DM-free survival stratified by FLI groups. The probability of surviving without developing T2DM varied considerably between FLI groups (log-rank test, *p* < 0.0001). The probability of surviving without T2DM rapidly fell as FLI increased, revealing the top group at greatest risk for developing T2DM.

### The results of the relationship between FLI and incident T2DM

The relationship between FLI and T2DM events was examined using the Cox proportional hazards regression model. In Table 4, we simultaneously displayed the three adjusted models and the non-adjusted models. FLI was found to be positively linked with incident T2DM in the crude model (HR = 1.041, 95% CI:1.038–1.045, *P* < 0.0001). The minimum adjusted model (adjusted DBP, age, SBP, ethanol consumption, sex, smoking status, exercise) did not show any discernible changes in the outcome (HR = 1.040, 95%CI: 1.035–1.045). A positive association between FLI and incident T2DM was also observed after accounting for the entire model (adjusted ALT, exercise, TC, age, HDL-C, AST, DBP, sex, SBP, alcohol intake, smoking status, glycated hemoglobin, fatty liver) (HR = 1.019, 95%CI: 1.012–1.025, *P* < 0.0001). The findings indicated that a unit rise in FLI raised the risk of T2DM by 1.9%.

**Table 4:** Relationship between FLI and the incident T2DM in different Cox proportional risk models

Variable	Crude model (HR,95%CI,P)	Model I(HR,95%CI,P)	Model II (HR,95%CI,P)	Model III (HR,95%CI,P)
FLI	1.041 (1.038, 1.045) < 0.00001	1.040 (1.035, 1.045) < 0.00001	1.019 (1.012, 1.025) < 0.00001	1.020 (1.013, 1.027) < 0.00001
FLI(quartile)				
Q1	Ref	Ref	Ref	Ref
Q2	1.819 (0.963, 3.434) 0.06513	1.547 (0.813, 2.944) 0.18333	1.307 (0.685, 2.495) 0.41617	1.261 (0.650, 2.446) 0.49336
Q3	4.854 (2.781, 8.472) < 0.00001	3.725 (2.069, 6.706) 0.00001	1.799 (0.979, 3.308) 0.05866	1.631 (0.854, 3.115) 0.13819
Q4	14.310 (8.476, 24.159) < 0.00001	10.371 (5.787, 18.588) < 0.00001	2.262 (1.171, 4.368) 0.01508	2.012 (1.003, 4.035) 0.04900
P for trend	< 0.00001	< 0.00001	0.00626	0.02514

Crude model:we did not adjust other covariants

Model I: we adjusted age, sex, SBP, DBP, ethanol consumption, smoking status and habit of exercise

Model II: we adjusted age, sex, SBP, DBP, ALT, AST, Glycated hemoglobin, TC, HDL-C, ethanol consumption, smoking status, exercise, fatty liver

Model III: we adjusted age(smooth), sex, SBP(smooth), DBP(smooth), ALT(smooth), AST(smooth), Glycated hemoglobin(smooth), TC(smooth), HDL-C(smooth), ethanol consumption(smooth), smoking status, exercise, fatty liver

*HR* Hazard ratios, *CI* Confidence, *Ref* Reference, *FLI* Fatty liver index, *T2DM* Type 2 diabetes mellitus

### Sensitivity analysis

The FLI was turned into a categorical variable for sensitivity analysis (Quartile). The findings of Model II revealed that the probability of acquiring T2DM rose with FLI quartiles 2, 3, and 4 compared to FLI quartile 1[HR 1.307 (0.685, 2.495), 1.799 (0.979, 3.308), and 2.262 (1.171, 4.368)]. In the entire model, the top quartile experienced a 1.26 times increase in T2DM risk compared to the bottom FLI quartile. And the T2DM risk trend in FLI quartiles was significant (P for trend = 0.00626) (Table 4).

In addition, we included the continuity covariate into the equation as a curve using a GAM. Model III results indicated the consistency of the findings with the fully adjusted model (HR = 1.020, 95%CI: 1.013–1.027, P < 0.00001), demonstrating the robustness of the findings (Table 4). The results of Model III also revealed that the probability of acquiring T2DM rose with FLI quartiles 2, 3, and 4 compared to FLI quartile 1[HR 1.261 (0.650, 2.446), 1.631 (0.854, 3.115), and 2.012 (1.003, 4.035)].

Furthermore, we calculated an E-value to evaluate the sensitivity to unmeasured confounding variables. The value of E was 1.16. The E-value was bigger than the relative risk of unmeasured confounders and incident T2DM, indicating that unmeasured or unknown confounders had minimal influence on the association between FLI and T2DM risk.

Furthermore, other sensitivity analyses excluded patients with fatty liver. After controlling for confounding factors, we found that the relationship between FLI and incident T2DM was not statistically significant in people without fatty liver (HR = 1.003, 95% CI:0.988 to 1.018). (Table 5). For sensitivity analysis, we additionally omitted any alcohol drinkers. After controlling for gender, SBP, ALT, DBP, TC, AST, HDL-C, smoking status, Glycated hemoglobin, exercise, age, and fatty liver, the results showed that FLI was still linked with incident T2DM (HR = 1.028, 95% CI:1.016 to 1.041, *P* < 0.00001) (Table 5). The findings of the sensitivity analysis revealed that the link between FLI and the risk of T2DM was very strong.

**Table 5:** Relationship between FLI and incident T2DM by Cox proportional risk models in different sensitivity analyses

Exposure	ModelI (HR,95%CI,P)	Model II (HR,95%CI,P)
FLI	1.003 (0.988, 1.018) 0.66641	1.028 (1.016, 1.041) < 0.00001
FLI (quartile)		
Q1	Ref	Ref
Q2	1.072 (0.539, 2.130) 0.84302	1.889 (0.632, 5.646) 0.25519
Q3	1.588 (0.800, 3.150) 0.18610	2.674 (0.916, 7.808) 0.07206
Q4	1.400 (0.626, 3.133) 0.41270	5.017 (1.561, 16.127) 0.00679
P for trend	0.31963	0.00269

Model I was sensitivity analysis after excluding those with fatty liver. We adjusted age, sex, ethanol consumption, smoking status, exercise, SBP, DBP, ALT, AST, TC, HDL-C, Glycated hemoglobin

Model II was sensitivity analysis after excluding any alcohol consumers. We adjusted age, sex, SBP, DBP, ALT, AST, TC, HDL-C, Glycated hemoglobin, smoking status, exercise, fatty liver

*HR* Hazard ratios, *CI* Confidence, *Ref* Reference, *FLI* Fatty liver index, *T2DM* Type 2 diabetes mellitus

### The results of subgroup analyses

We used subgroup analysis to take into account other influencing factors, such as SBP, that could affect the findings on the connection between FLI and incident T2DM. We used FPG, exercise, age, ethanol consumption, fatty liver, sex, smoking status, SBP and DBP as the stratification variables to detect the trend of effect sizes in these variables (Table 6). Table 6 showed that exercise and ethanol consumption could modify the relationship between FLI and incident T2DM (P for interaction < 0.05). And a stronger association between FLI and incident T2DM was observed in the regular exercisers (HR = 1.036, 95%CI: 1.019–1.053, *P* < 0.0001) and the population without ethanol consumption (HR = 1.028, 95%CI: 1.017–1.039, *P* < 0.0001). In contrast, the weaker association between FLI and incident T2DM was probed in the people who was consuming alcohol (HR = 1.015, 95%CI:1.008, 1.022) and not exercising regularly (HR = 1.016, 95%CI:1.009, 1.023). These findings revealed a robust association between FLI and incident T2DM in the majority of subgroups.

**Table 6:** Effect size of FLI on T2DM in prespecified and exploratory subgroups

Characteristic	No of participants	HR (95%CI)	*P* value	P for interacion
Age, years				0.9707
< 30	402	1.093 (0.000, Inf)	0.9998	
30 to 40	4892	1.022 (1.008, 1.037)	0.0024	
40 to 50	5293	1.017 (1.007, 1.028)	0.0006	
50 to 60	3057	1.016 (1.002, 1.030)	0.0215	
≥ 60	636	1.023 (0.992, 1.056)	0.1534	
Sex				0.4099
Male	7440	1.018 (1.011, 1.025)	< 0.0001	
Female	6840	1.023 (1.011, 1.036)	0.0002	
FPG, mmol/L				0.2733
< 5.1	6383	1.030 (1.009, 1.052)	0.0054	
≥ 5.1	7897	1.018 (1.011, 1.024)	< 0.0001	
Regular exercisers				0.0313
Yes	2476	1.036 (1.019, 1.053)	< 0.0001	
No	11,804	1.016 (1.009, 1.023)	< 0.0001	
SBP, mmHg				0.2172
< 140	13,619	1.021 (1.014, 1.028)	< 0.0001	
≥ 140	661	1.011 (0.997, 1.025)	0.1222	
DBP, mmHg				0.1964
< 90	13,640	1.020 (1.013, 1.027)	< 0.0001	
≥ 90	640	1.008 (0.992, 1.025)	0.3300	
Smoking status				0.2437
Non-smoker	8751	1.023 (1.014, 1.033)	< 0.0001	
Ex-smoker	2572	1.008 (0.993, 1.024)	0.2732	
Current smoker	2957	1.019 (1.010, 1.029)	< 0.0001	
Ethanol consumption, g/week				0.0359
= 0	4735	1.028 (1.017, 1.039)	< 0.0001	
> 0	9545	1.015 (1.008, 1.022)	< 0.0001	
Fatty liver				0.0853
No	11,765	1.009 (0.996, 1.023)	0.1749	
Yes	2515	1.022 (1.015, 1.030)	< 0.0001	

Note 1:Above model adjusted for age, sex, SBP, DBP, ALT, AST, Glycated hemoglobin, TC, HDL-C, fatty liver, ethanol consumption, smoking status and exercise

Note 2:In each case, the model is not adjusted for the stratification variable

*HR* Hazard ratios, *CI* Confidence interval, *DBP* Diastolic blood pressure, *FPG* Fasting plasma glucose, *SBP* Systolic blood pressure, *T2DM* Type 2 diabetes mellitus

### The results of the ROC curve analysis

**Fig. 5:**
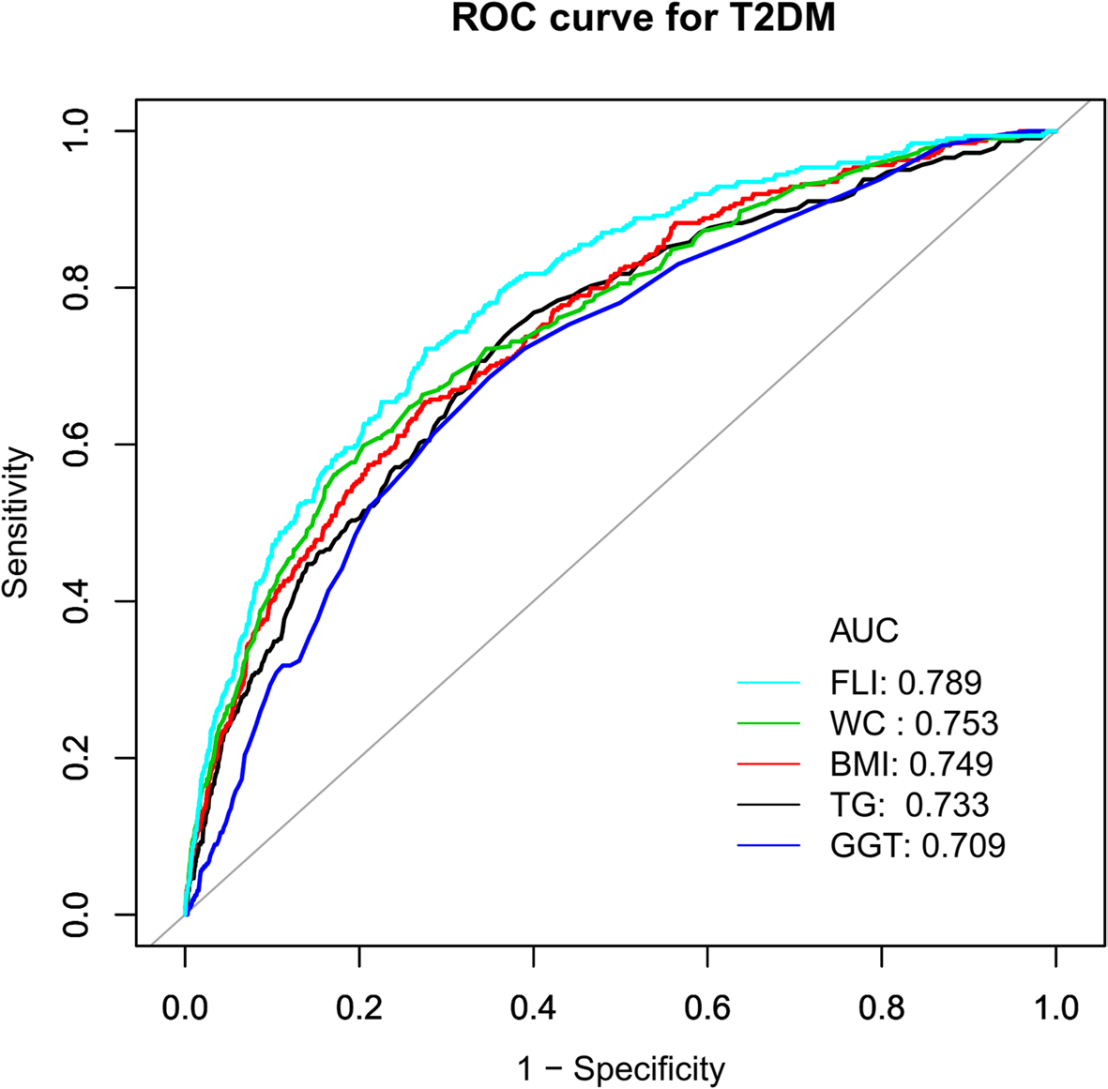
The results of ROC curve analysis for measuring the ability of FLI, WC, BMI, TG, and GGT to predict the risk of T2DM. The areas under the curve (AUC) of each variable were as follows: GGT: 0.709 < TG: 0.733 < BMI: 0.749 < WC: 0.753 < FLI: 0.789

In addition, we drew a ROC curve to measure the ability of FLI, WC, BMI, TG, and GGT to predict the risk of T2DM (Fig. 5). The areas under the curve (AUC) of each variable were as follows: GGT: 0.709 < TG: 0.733 < BMI: 0.749 < WC: 0.753 < FLI: 0.789 (Table 7). The highest Youden index of GGT, TG, BMI, WC, and FLI was 0.3365, 0.3711, 0.3792, 0.3941, 0.4463, and the corresponding optimal cut-off value was 17.5000, 0.8637, 23.5285, 83.1500, 13.5821, respectively. Besides, we calculated the net reclassification improvement (NRI) and integrated discrimination improvement (IDI) to compare the predictive values among the different models. Compared with WC, the IDI and NRI of the FLI were all increased(Table S1). Combining the AUC, Youden index, IDI, and NRI, we concluded that the predictive ability of FLI to incident T2DM was better than that of other variables (Table 7, Table S1).

**Table 7:** Predictive value FLI, WC, TG, BMI, and GGT for predicting T2DM

Variables	AUC	95% CI lower bound	95% CI upper bound	Best threshold	Specificity	Sensitivity	Youden Index
TG	0.7330	0.7048	0.7611	0.8637	0.6242	0.7469	0.3711
BMI	0.7491	0.7220	0.7761	23.5285	0.7249	0.6543	0.3792
WC	0.7535	0.7258	0.7812	83.1500	0.7953	0.5988	0.3941
GGT	0.7091	0.6815	0.7368	17.5000	0.6513	0.6852	0.3365
FLI	0.7895	0.7647	0.8143	13.5821	0.7241	0.7222	0.4463

*AUC* Area under the curve, *BMI* Body mass index, *CI* Confidence interval, *FLI* Fatty liver index, *GGT* Gammaglutamyltransferase, *TG* Triglyceride, *T2DM* Type 2 diabetes mellitus, *WC* Waist circumference

## Discussion

Our findings demonstrated that FLI was positively linked with the development of T2DM after controlling for other variables. Moreover, we conducted subgroup analysis to better understand the trend of FLI and the incidence of T2DM in different populations. Our study detected a stronger association in people with regular exercise and the population without ethanol consumption. On the contrary, a weaker relation between FLI and incident T2DM was observed in people who were consuming alcohol and not exercising regularly.

FLI is a simple and valuable clinical biomarker of hepatic steatosis because it only requires simple biochemical (GGT and TG) and anthropometric (WC and BMI) measurements. So FLI had been adopted by the European Association for the Study of the Liver (EASL), the European Association for the Study of Diabetes (EASD), and the European Association for the Study of Obesity (EASO) NAFLD guidelines as the preferred diagnostic tool for larger-scale screening studies [33]. It is accurate in detecting fatty liver. This index could also replace ultrasonography to diagnose the degree of liver steatosis [12]. Several preceding studies had proposed that FLI could predict the risk of diabetes [34–40], which agreed with our conclusions. Moreover, we constructed a ROC curve to estimate the ability of WC, BMI, TG, FLI, and GGT to predict the risk of T2DM, and we found that FLI’s AUC and Youden index was better than any other component of FLI.

Researchers have clarified the associations between FLI and the risk of diabetes [39, 40]. Several previous studies mentioned that high FLI increases the risk of developing diabetes mellitus [41–43]. For instance, FLI > 60 is independently related to the occurrence of T2DM after three years of follow-up in the Spanish PREDAPS study regardless of sex, age, or education level [44]. A recently published meta-analysis[16]with a sample of 70,918(27 studies) participants reported a direct relationship between the highest grade of FLI and an increased diabetes risk, which was not affected by continent, sex, and the quality of study after subgroup analysis. Besides, Wargny et al. [45] found that low FLI value was independently related to prediabetes reversion. FLI strongly predicted the risk of diabetes in a 5-year, prospective, observational study of 389 individuals with prediabetes, independently of traditional risk factors for diabetes mellitus such as sex, age, FPG, or glycated hemoglobin. We obtained a similar result from the Cox proportional hazard regression model, suggesting a significant and robust association between FLI and incident T2DM. It was worth noting that most participants in the present study had an FLI of less than 60. Firstly, the Japanese population might have different distribution of FLI compared to Caucasian populations due to different anthropometric characteristics. In addition, this study excluded participants with FPG > 6.1 mmol/L and heavy drinking (ethanol consumption of more than 30 g per day for men and 20 g per day for women). While this study also found that alcohol consumption and FPG increased with the increase in FLI. In addition, we found that they were also risk factors for diabetes in the present study. However, the PREDAPS study found FLI was independently associated with the incidence of T2DM when it was above 60 [44]. Obviously, the baseline alcohol consumption and FPG levels in the PREDAPS study were higher than those in the present study because their study population was pre-diabetes participants. Incident diabetes was positively associated with FLI, according to our research. The sensitivity analysis, however, revealed that this association is still present among those who do not use alcohol. Despite the majority of research participants having FLI levels below 60, the results served as a guide for treatment interventions to lower the risk of diabetes. This discovery would further expand the results of the PREDAPS study.

Surprisingly, by univariate Cox proportional hazard model, we found that exercise was not significantly associated with T2DM. However, as can be seen in Table 3, although exercise was not significantly associated with T2DM, there was a trend to decrease the risk of T2DM. Moreover, we did a subgroup analysis, and the interaction test was statistically significant. We found that in the population with regular exercise, the relationship between FLI and T2DM was enhanced. The reason might be that in patients with exercise, the level of other diabetes risk factors such as BMI and lipid profile decreased, so the effect of FLI on diabetes was relatively enhanced.

There are some strengths in our study. First, compared with most previous studies of the same kind, our sample size is relatively large. Second, since this study was observational, it was susceptible to potential confounding factors. We made rigorous statistical adjustments to reduce the interference of confounding factors to ensure the reliability of the results. Third, to ensure the validity of the findings, we performed a number of sensitivity analyses. These included categorizing the FLI, using a GAM to incorporate the continuity covariate as a curve into the equation, calculating E-values to investigate the possibility of unmeasured confounding, and re-examining the relationship between FLI and incident T2DM after excluding alcohol consumers and participants with fatty liver. Fourth, the effect modifier factor analysis allowed us to use the data better and draw conclusions steadily in the different subgroups of our study. Fifth, to assess the accuracy of the FLI, TG, WC, GGT, and BMI in predicting the risk of T2DM, we created a ROC curve.

There are some limitations in the current study because of the original study [17]. First, since all subjects are of Japanese descent, this study is not generalisable to all population. More research is required to ascertain the prevalence of diabetes, the relationship between FLI and diabetes, and more in people with various genetic backgrounds. The findings of this study might not be generalizable, given that subjects with a significant drinking habit, viral hepatitis, or any medication usage at baseline were omitted. Second, because this study is based on a secondary analysis of previously published data, it is not possible to make adjustments for variables like insulin concentration, low-density lipoprotein cholesterol, very low-density lipoprotein cholesterol, tumor necrosis factor, and interleukin-6 that were not part of the original dataset. To evaluate the possible impact of unmeasured confounders, we estimated the E-value and observed that unmeasured confounders were unlikely to explain the results. Third, censoring by death during follow-up was not included in the data from the initial study. Possible participant fatalities during follow-up were unavoidable for such a large sample of subjects. We can plan our research and gather information on potential future death censorship. So, using a competitive risk model, we may examine the connection between FLI and diabetes. Fourth, due to the lack of oral glucose tolerance tests, the incidence of diabetes may be underestimated. However, due to practical reasons and logistics, performing oral glucose tolerance tests on all participants is not feasible. Fifth, the study used ultrasonography that could not detect steatohepatitis (SH), which diagnosis could be obtained only by biopsy [12]. However, for obvious ethical reasons, an SH score will never be available in a representative sample of the general population. We can use other non-invasive scores to diagnose NAFLD for further studies, such as NAFLD Fibrosis score, Hepatic Steatosis index, FIB-4, APRI, lipid accumulation product. Sixth, the FLI and other parameters were only assessed at baseline in the current investigation, and FLI changes over time, information on first-line laboratory tests and repetition were not considered. In the future, we can think about structuring our studies or working with other researchers to gather as many data points as possible, such as details on the development of FLI during patient follow-up. Finally, rather than establishing a causal relationship between FLI and diabetes risk, this retrospective observational analysis presented an association inference.As a result, our findings should be interpreted with caution and confirmed by future research.

## Conclusion

This study found a positive connection between FLI and incident T2DM in the Japanese population. As a result, aberrant FLI assists in identifying the Japanese population at high risk of T2DM, which would allow doctors to plan and implement suitable care methods ahead of time. And it would allow people to change their lifestyles in advance to lower the occurrence of T2DM by reminding them of the increased risk of diabetes during follow-up.

## Supplementary Information

**Additional file 1.** Data.

**Additional file 2:** **Table S1.** Model performance of FLI compared with WC. **Figure S1.** Data visualization of FLI of all participants from the T2DM and non-T2DM groups.

## Data Availability

Data can be downloaded from the ‘DATADRYAD’ database (www.Datadryad.org). All data generated or analysed during this study are included in the supplementary information files.

## Abbreviations

ALT: Alanine aminotransferase
AUC: Area under the curve
AST: Aspartate aminotransferase
DBP: Diastolic blood pressure
BMI: Body mass index
FPG: Fasting plasma glucose
FLI: Fatty liver index
GAM: Generalized additive models
GGT: Gammaglutamyltransferase
HDL-C: High-density lipoprotein cholesterol
IDI: Integrated discrimination improvement
IR: Insulin resistance
NRI: Net reclassification improvement
ROC: Receiver operating characteristic
SBP: Systolic blood pressure
SH: Steatohepatitis
T2DM: Type 2 diabetes mellitus
TC: Total cholesterol
TG: Triglyceride
WC: Waist circumference
