# Supplementary material for: The association between fatty liver index and onset of diabetes: secondary analysis of a population-based cohort study
Source: BMC Public Health. 2023 Apr 11;23:679. doi: 10.1186/s12889-023-15442-z (PMC10091632; doi:10.1186/s12889-023-15442-z)
Supplement: Supplementary file 2 — Additional file 2: Table S1. Model performance of FLI compared with WC. Figure S1. Data visualization of FLI of all participants from the T2DM and non-T2DM groups. [file 12889_2023_15442_MOESM2_ESM.docx]

**The association between fatty liver index and onset of diabetes: secondary analysis of a population-based cohort study**

**Running title: Fatty liver index and incident diabetes mellitus**

**Yanqiong Zhu^1#^, Haofei Hu^2,3,4#^, Yumei Wu^5^, Yinhua Rao^6^, Qixiang Li^1^, Xuehui Duan^1^, Guopeng Yao ^1^, Hekun Yin^1*^, Ziyi Luo^5*^**

^1^Department of Gastroenterology, Jiangmen Central Hospital, Jiangmen 529030, Guangdong Province, China

^2^Department of Nephrology, The First Affiliated Hospital of Shenzhen University, Shenzhen 518000, Guangdong Province, China

^3^Department of Nephrology, Shenzhen Second People’s Hospital, Shenzhen 518000, Guangdong Province, China

^4^Shenzhen University Health Science Center, Shenzhen 518000, Guangdong Province, China

^5^Department of Gastroenterology, Shenzhen Third People's Hospital, Shenzhen 518000, Guangdong Province, China

^6^Department of Gastroenterology, Shenzhen People's Hospital Longhua Branch, Shenzhen 518000, Guangdong Province, China

**^#^**Yanqiong Zhu and Haofei Hu have contributed equally to this work.

*Corresponding author

Ziyi Luo,

Shenzhen Third People's Hospital,

No.29 Bulan Road, Longgang District,

Shenzhen 518000,

Guangdong Province,

China,

Tel:+86-13827101707,

E-mail:1731360398@qq.com

*Corresponding author

Hekun Yin,

Department of Gastroenterology,

Jiangmen Central Hospital,

No.23 Haibang Street, Pengjiang District

Jiangmen 529030,

Guangdong Province,

China,

Tel:+86-18320327808,

E-mail: [hekunyin@163.com](mailto:hekunyin@163.com)

**Table S1. Model performance of FLI compared with WC**

| Variables | WC | FLI |
| --- | --- | --- |
| AUC | 0.754 (0.726 to 0.781) | 0.790(0.765 to 0.814) |
| P-value | - | <0.0001 |
| NRI | - | 0.0491 (0.0093 to 0.0889) |
| P-value | - | 0.0155 |
| IDI | - | 0.0491 (0.0093 to 0.0890) |
| P-value | - | 0.0157 |

AUC area under the curve; NRI, net reclassification improvement; IDI, integrated discrimination improvement; FLI, fatty liver index; WC, waist circumference

FigureS1. Data visualization of FLI of all participants from the T2DM and non-T2DM groups.
